# Deep intracochlear injection of triamcinolone-acetonide with an inner ear catheter in patients with residual hearing

**DOI:** 10.3389/fnins.2023.1202429

**Published:** 2023-07-26

**Authors:** Nils K. Prenzler, Rolf Salcher, Thomas Lenarz, Lutz Gaertner, Anke Lesinski-Schiedat, Athanasia Warnecke

**Affiliations:** ^1^Department of Otorhinolaryngology, Head and Neck Surgery, Hannover Medical School, Hanover, Germany; ^2^Cluster of Excellence “Hearing 4 All” (DFG Exc. 2177), Hannover Medical School, Hanover, Germany

**Keywords:** cochlear implant, hearing preservation, impedances, steroids, catheter, drug delivery, inner ear

## Abstract

**Introduction:**

In a previous study, an inner ear catheter was used to deliver low- and high-dose steroids into the cochlea prior to cochlear implant electrode insertion. With this approach, more apical regions of the cochlea could be reached and a reduction of electrode impedances in the short term was achieved in cochlear implant recipients. Whether intracochlear application of drugs via the catheter is a safe method also for patients with residual hearing has not been investigated hitherto. The aim of the present study was therefore to investigate the effect of intracochlear triamcinolone application in cochlear implant recipients with residual hearing.

**Patients and methods:**

Patients with residual hearing were administered triamcinolone-acetonide (4 mg/ml; *n* = 10) via an inner ear catheter just prior to insertion of a MED-EL FLEX28 electrode. Impedances were measured at defined time points (intra-operatively, post-operatively and at first fitting) and retrospectively compared with a control group (no steroid application) and low- and high-dose group. Hearing thresholds were measured preoperatively, 3 days after surgery and at first fitting by pure tone audiometry. Pre- to postoperative hearing loss was determined at first fitting and compared to results from a previous study.

**Results:**

The median hearing loss after implantation (125–1,500 Hz) was 20.6 dB. Four patients (40%) showed a median hearing loss of less than 15 dB, three patients (30%) between 15 and 30 dB and three patients (30%) more than 30 dB. The median hearing loss was similar to the results obtained from our previous study showing a median hearing loss of 24 dB when using FLEX28 electrode arrays.

**Conclusion:**

No difference in residual hearing loss was found when comparing application of triamcinolone-acetonide using an inner ear catheter prior to the insertion of a FLEX28 electrode array to the use of the FLEX28 electrode array without the catheter. Thus, we conclude that application of drugs to the cochlea with an inner ear catheter could be a feasible approach in patients with residual hearing.

## 1. Introduction

Hearing loss is the most common sensorineural disorder with a devastating impact on the quality of life (Dixon et al., 2020). If not treated sufficiently, patients with hearing loss are at increased risk to develop comorbidities such as depression or dementia (Livingston et al., 2017). Thus, effective and early treatment is mandatory (Bisogno et al., 2021). Patients with severe hearing loss are treated with cochlear implantation to bypass damaged sensory hair cells and to directly activate auditory neurons. With dramatic technological progress in the last decades and improved surgical skills, the indication criteria for cochlear implantation have been broadened and even patients with significant residual hearing are nowadays candidates for cochlear implantation (Lenarz, 2017). It is estimated that approximately 80% of cochlear implant recipients have bilateral residual hearing in the lower frequencies (Sheffield et al., 2015). Preserved residual hearing in the lower frequencies is among a few other factors the most important discriminator between good and poor performers (Gantz et al., 2022). Preserving residual hearing offers the possibility to combine acoustic and electrical stimulation. This combined stimulation technique leads to a complementary improvement in speech perception, especially in noisy situations and for complex languages like Mandarin (Adunka et al., 2008; Dorman and Gifford, 2010; Sheffield et al., 2015; Sargsyan et al., 2021; Schaefer et al., 2021; Li et al., 2022). Unfortunately, a significant portion of these patients lose their residual hearing after implantation (Maria et al., 2014). If it occurs within the perioperative time, hearing loss might be related to direct intracochlear surgically induced trauma. With biotechnological and pharmaceutical advances, future regenerative therapies targeting the inner ear are emerging (Schilder et al., 2018, 2019) and require cochlear structure preservation. Thus, protection of the ultrastructural architecture of the cochlea including their neuronal connections is important for sustained preservation of residual hearing.

Pharmacological treatment to prevent loss of residual hearing includes the administration of steroids via different modalities, i.e., systemic, intratympanic, and intracochlear (Salt and Plontke, 2009; Parys et al., 2022). Systemic delivery is easy to perform and has been the mainstay for treating sudden sensorineural hearing loss and to preserve residual hearing during cochlear implantation (O’Leary et al., 2021; Skarzynska et al., 2021). Despite this fact, there are several disadvantages associated with systemic delivery of drugs to the inner ear: first pass effect, not sufficient for every drug due to the blood-labyrinth barrier and an increased risk of systemic adverse effects (Parys et al., 2022). In addition, ineffective concentrations of the drug inside the cochlea may be reached after systemic application (Bird et al., 2011). Transtympanic drug delivery can overcome many of the disadvantages of systemic application (Parys et al., 2022) and higher perilymph concentrations of the steroid have been measured after transtympanic administration of dexamethasone when compared to systemic application (Bird et al., 2011). However, how much of the applied drug diffuses through the round window into the inner ear cannot be controlled (Parys et al., 2022). Most importantly, a limited diffusion has been observed in the inner ear and many of the drugs do not reach the medial and apical regions of the cochlea after transtympanic application (Plontke et al., 2008; Salt and Plontke, 2009). In addition, anatomical variations as well as the inherent and individual permeability of the round window membrane result in an insufficient dose accumulation of the applied drug (Plontke et al., 2017). Intracochlear administration of drugs can be realized by direct single shot injection. For more sustained drug release, drug-eluting electrodes or electrodes connected to a catheter and an osmotic pump enable an intrascalar delivery up to the medial turn of the cochlea (Plontke et al., 2017; Manrique-Huarte et al., 2020; Dhanasingh and Hochmair, 2021). Another method to provide the cochlea locally with drugs prior to cochlear implantation is the use of an inner ear catheter as a drug delivery device (Prenzler et al., 2018; Yildiz et al., 2023). It has been successfully used for glucocorticoid delivery in preclinical models for preservation of residual hearing (Ibrahim et al., 2010; Ibrahim et al., 2011). This application method allows a defined quantity of substances to be applied to a defined location within the cochlea. Until now, this had not been possible either through systemic or any other local drug delivery strategy. However, this approach requires the insertion of the catheter to the cochlea and a subsequent second insertion of the electrode array (Prenzler et al., 2018; Yildiz et al., 2023). There have been concerns rising that this procedure may not be suitable for patients with residual hearing since it may increase the risk of loss of residual hearing due to additional mechanical trauma by the catheter insertion. The aim of the present study was therefore to evaluate the safety of sequential catheter application followed by electrode insertion in patients with residual hearing.

## 2. Patients and methods

**Figure 1:**
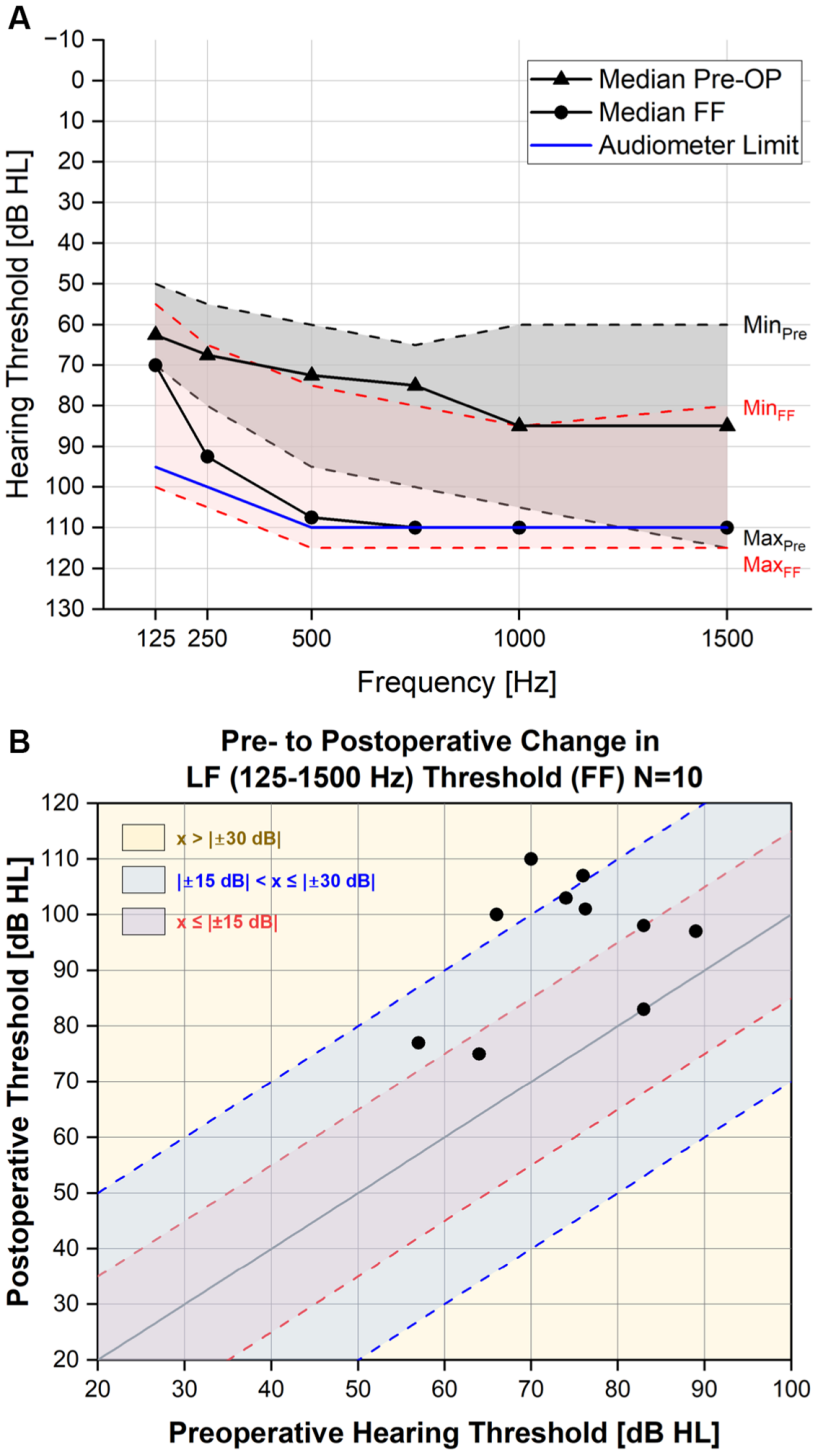
**(A)** Pure tone average pre- and post-operatively. The threshold levels of the PTA for each frequency in the lower range (125–1,500 Hz) are depicted here with minimum and maximum values included. **(B)** Individual pre- to post PTA shift in the low frequencies (one point indicates an individual subject).

Ten adult patients (age ranging from 36.9–86.5 years (mean 59 ± 15 STD (standard deviation)), 4 female, 6 male) were included in the retrospective analysis. All patients underwent a CI surgery with an indication for a Flex28 electrode array. They had preoperative residual hearing in the low frequencies with a median low-frequency air-conduction threshold averaged across the frequencies (125, 250 Hz, 500, 1,000 and 1,500 Hz) of 75 dB HL (min: 57 dB HL; max: 89 dB HL) (see also Figure 1A). For all ten patients it was decided on an individual basis to apply triamcinolone-acetonide with an inner ear catheter (now CE- certified and at the time point of the study a custom-made device from MED-EL, Innsbruck, Austria) prior to electrode insertion to control inflammation associated with implantation trauma.

All patients were prepared for cochlear implantation according to our standard as follows: performing the mastoidectomy, drilling of the implant bed, performing the posterior tympanotomy, drilling the bony notch for fixating the electrode array at the chorda-facial angle and exposure of the round window membrane. Thereafter, the catheter was connected to a 1 mL syringe containing a triamcinolone-acetonide solution at a concentration of 20 mg/mL and preloaded with the steroid [Triamhexal ® 40 mg/ml (Hexal, Holzkirchen, Germany)] diluted at a ratio of 1:9 with Ringer solution (Braun, Melsungen, Germany). For detailed information on the inner ear catheter, the reader is referred to our previous publications (Prenzler et al., 2018, 2020). The round window was then opened with a sharp needle followed by a slow manual insertion of the prepared inner ear catheter. After achieving maximal insertion (20 mm), a very slow manual injection of the triamcinolone-acetonide solution followed until a backflush of the white fluid was observed at the round window. The catheter was then removed very slowly and the Flex28 electrode array was inserted as performed usually. Since insertion, injection and removal of the catheter have been performed manually, the pace cannot be quantified.

Until the first fitting appointment (approximately 6 weeks post-op), no audio processor was worn. To document hearing preservation, the hearing loss after cochlear implantation (PTA shift; pure tone average shift) was determined by subtraction and subsequent averaging of the preoperative air conduction threshold the frequencies 125, 500, 1,000 and 1,500 Hz from the threshold measured at first fitting using pure-tone audiometry. For the measurements, patients were seated in a sound proof chamber and instructed to press the button immediately when hearing a pure tone. The contralateral ear was plugged and muffled when necessary. Pure-tone audiometer limits were 95 dB at 125 Hz, 100 dB at 250 Hz, and 110 dB at 500 to 1,500 Hz. If no hearing could be measured up to the audiometer limit, the thresholds were set to audiometer limit +5 dB. This is a best-case assumption and corresponds to the audiometer limit + the minimum audiometer step size. If hearing thresholds for a specific frequency was not measurable preoperatively, this frequency was not considered for the calculation of the PTA shift. A change in the preoperative to postoperative air conduction was evaluated arithmetically.

Impedances were measured for each electrode contact intraoperatively (intra-OP), postoperatively (3 days), at the first fitting before activation (FF) and after activation of the device electrode (FF-el). Electrode impedances were obtained using the standard MED-EL telemetry system (MAX interface box, clinical software Maestro 68).

The impedance results were compared to our previous results obtained from patients, who received a deep intracochlear administration of triamcinolone-acetonide at a concentration of 4 mg/mL (“low-dose”) prior to insertion of the Flex28 electrode (Prenzler et al., 2018). As a control, we used the control group of our previous publication (40–81 years old, one female, four male; no local steroid treatment) (Prenzler et al., 2018).

All data were pseudonymized prior to analysis.

To classify hearing preservation, the pre-to postoperative low-frequency hearing loss was clustered as previously reported (Suhling et al., 2016): PTA shifts ≤15 dB: good hearing preservation; PTA shifts >15 to ≤30 dB: moderate hearing preservation and PTA shifts >30 dB: poor hearing preservation. This procedure allows comparability to our previous results achieved with the MED-EL Flex electrode arrays.

Impedance measurements of all 12 electrode contacts were averaged per patient for every time point for each impedance value. To assess different regions of the cochlea, the electrode contacts were furthermore clustered and averaged as follows: apical (C1–C5), medial (C6–C8), and basal (C9–C12). The clustering of the electrode contacts was performed accounting not only the different regions that are covered intra-cochlearly but also the fact that C1–C5 in the Flex28 electrode array are single (on one side) and C6–C12 are double electrode contacts (on both sides). Thus, contact C5 was added to the apical contacts, since the single sided contacts might *per se* exhibit different impedance values. The three groups (CC; catheter, CC-HP; catheter-HP, control) were treated as independent samples. To test for equality of variances, the Levene’s test was used. One-way analysis of variance (ANOVA) was used to test for significant differences among the groups. No post-hoc test was performed as no significant difference was detected between the groups. *p* < 0.05 were considered significant. All data were analyzed statistically using IBM SPSS Statistics 22.

## 3. Results

As in our previous studies, none of the patients enrolled in this analysis experienced any perioperative complications. All patients included in the study showed a correct position of the electrode array inside the cochlea as determined by postoperative cone beam CT scans.

### 3.1. Hearing thresholds and hearing preservation

The median pre- and postoperative (first fitting appointment) air-conduction thresholds up to 1.5 kHz are shown in Figure 1A and the individual pre- to postoperative change in PTA (125–1,500 Hz) are shown in Figure 1B. The median PTA shift for the CC-HP group (*n* = 10) was 20.6 dB (Table 1). The majority of the patients (70%) showed a threshold shift of up to 30 dB at first fitting. Four patients showed hearing loss of 15 dB or less (40%) and three patients between more than 15 and less than 30 dB (30%). The remaining patients showed a hearing loss of more than 30 dB, but only one of those lost the complete residual hearing. For better comparability to our previous results, we included the results of Suhling et al. in Table 1 (Suhling et al., 2016).

**Table 1:** Hearing preservation rates at first fitting compared to our previously published results.

	Median PTA shift (125–1,500 Hz)	PTA shift (125–1,500 Hz)		
		≤15 dB	>15 – ≤ 30 dB	>30 dB
CC-HP (FLEX28) (*n* = 10)	20.6 dB	4 (40%)	3 (30%)	3 (30%)
FLEX20 (*n* = 46)	17.5 dB	21 (45.6%)	12 (26.1%)	13 (28.3%)
FLEX24 (*n* = 34)	20 dB	10 (29.4%)	18 (52.9%)	6 (17.7%)
FLEX28 (*n* = 40)	24 dB	6 (15.0%)	20 (50.0%)	14 (35.0%)

First column: Median PTA shift in low and medial frequencies (125, 250, 500, 1,000, 1,500 Hz) from patients treated with an intracochlear triamcinolone suspension (4 mg/mL) by use of an inner ear catheter directly before insertion of a Flex28 electrode [CC-HP (FLEX28)] compared to data from Suhling et al. (2016) patients with different lengths of implanted Flex-electrodes (20, 24, and 28 mm). Second to fourth column: total and proportion of patients from the different groups who achieved good (PTA shift ≤15 dB), moderate (15 < PTA shift ≤30 dB) or poor (PTA shift >30 dB) hearing preservation.

### 3.2. Impedance measurements

**Figure 2:**
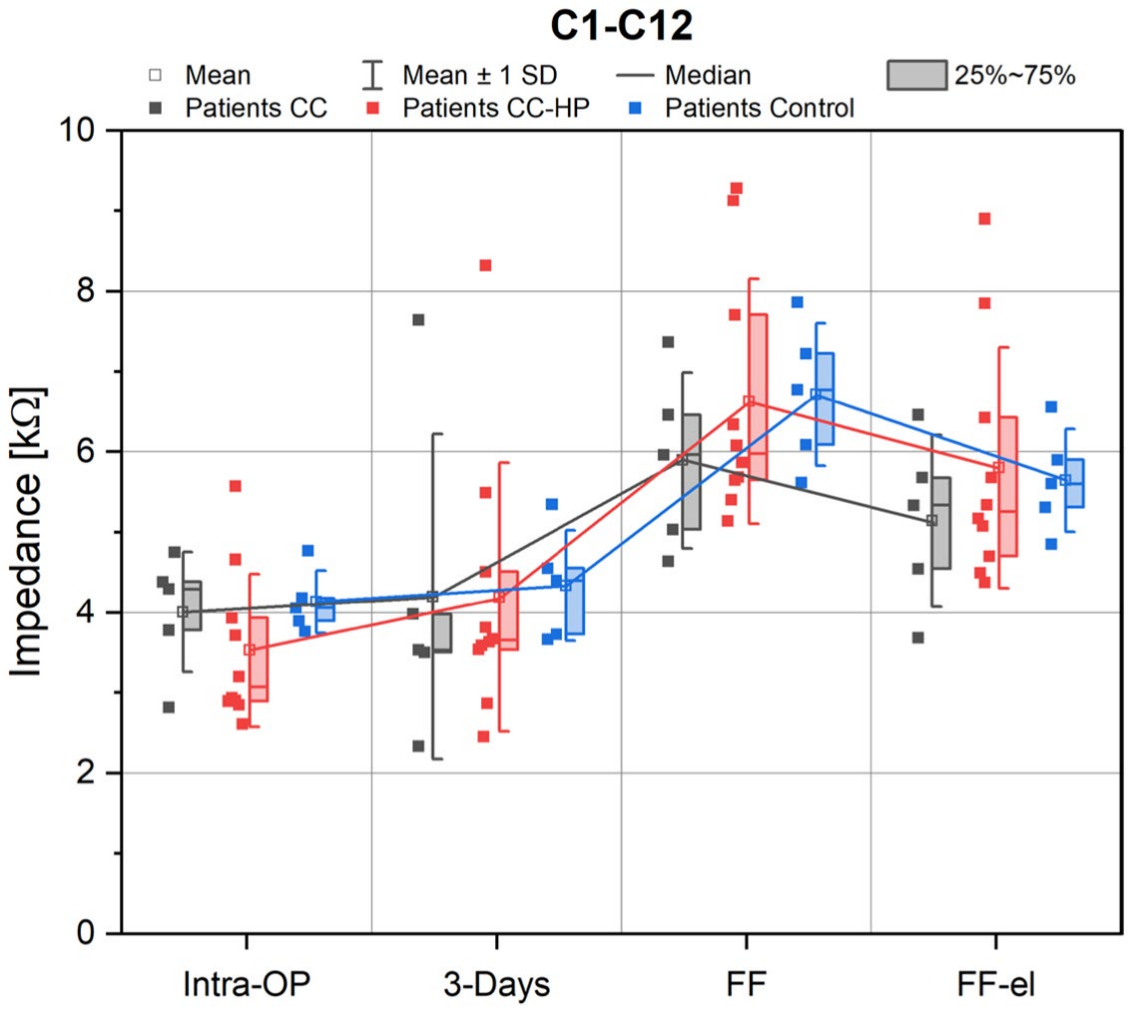
Depicted are the impedance values of the patients with residual hearing (CC-HP) in comparison to a control group and to patients without residual hearing treated with the inner ear catheter and steroid application as published earlier (Prenzler et al., 2018). Impedance values are shown as measured intraoperatively (Intra-OP), 3 days after surgery, at the first fitting (FF) as well as at the first fitting after electrical stimulation (FF-el).

A rise of the impedances at the first fitting prior to electrode activation was observed for all groups. After initial activation of the electrode, impedance values decreased immediately (FF-el) for all groups. A similar behavior of the impedances was also observed for the control group (patients treated with the FLEX28 electrode array without any steroids and without the use of the catheter) (Figure 2).

No significant difference was found between the three groups for the mean impedances across all electrodes (C1-C12) for the observed appointments [ANOVA: Intra-OP: *F*(2,17) = 1.156, *p* = 0.338; 3 days: *F*(2,17) = 0.015, *p* = 0.985; FF: *F*(2,17) = 0.648, *p* = 0.536; FF-el: *F*(2,17) = 0.476, *p* = 0.629].

**Figure 3:**
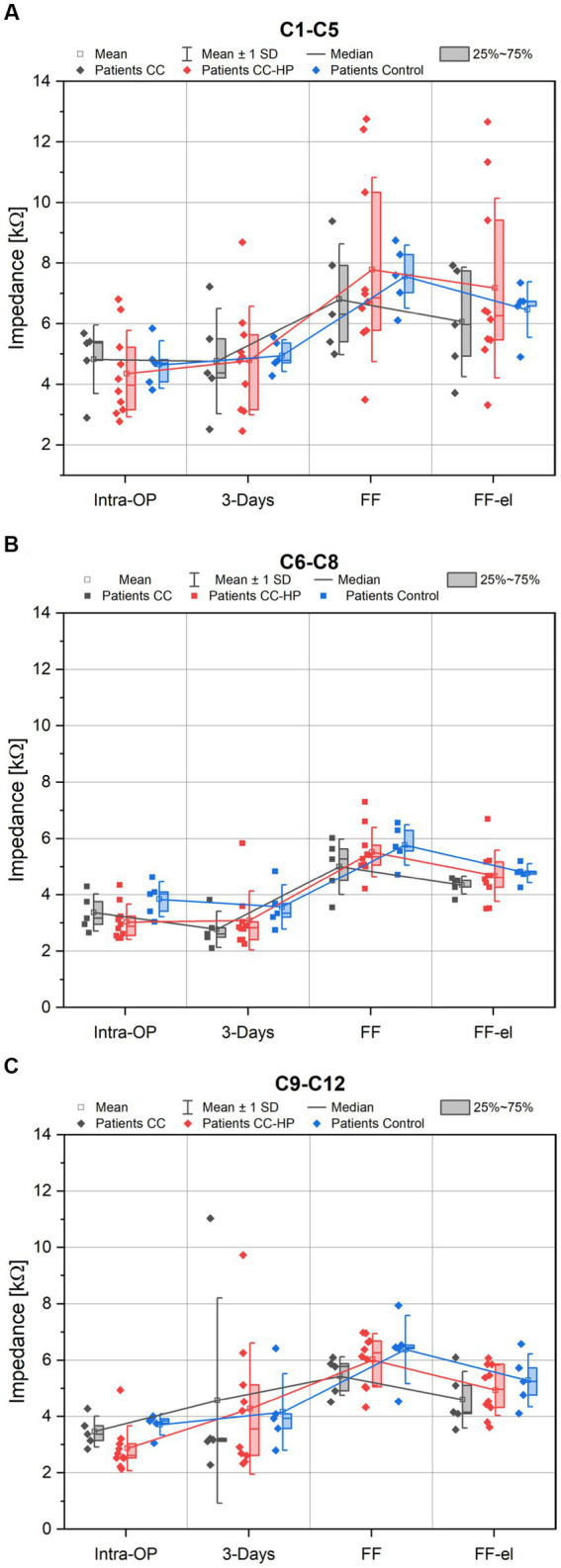
Depicted are the impedance values of the patients with residual hearing (CC-HP) in comparison to a control group and to patients without residual hearing treated with the inner ear catheter and steroid application as published earlier (Prenzler et al., 2018). Only impedance values of the apical electrode contacts (C1-C5) are shown in **(A)**, Only impedance values of the medial electrode contacts (C6-C9) are shown in **(B)**, Only impedance values of the basal electrode contacts (C9-C12) are shown in **(C)**.

Individual analysis for the different regions of the cochlea (basal, medial and apical) were also performed. For the basal region (electrode contacts 9–12), the course of the mean impedances was similar to the means over all electrode contacts in all groups (Figures 3A–C). When analyzing the medial contacts (C6–C8) and the apical contacts (C1–C5) only, a similar behavior of the impedances was observed (Figure 3).

There were no significant differences found between control group and the two catheter groups for the different regions of the cochlea: apical region (C1–C5): [ANOVA: Intra-OP: *F*(2,17) = 0.269, *p* = 0.767; 3 days: *F*(2,17) = 0.021, *p* = 0.979; FF: *F*(2,17) = 0.274, *p* = 0.764; FF-el: *F*(2,17) = 0.410, *p* = 0.670], medial region (C6–C8) [ANOVA: Intra-OP: *F*(2,17) = 1.777, *p* = 0.199; 3 Days: *F*(2,17) = 0.994, *p* = 0.391; FF: *F*(2,17) = 1.049, *p* = 0.372; FF-el: *F*(2,17) = 0.531, *p* = 0.597] and basal region (C9–C12) [ANOVA: Intra-OP: *F*(2,17) = 3.093, *p* = 0.072; 3 Days: *F*(2,17) = 0.035, *p* = 0.966; FF: *F*(2,17) = 1.265, *p* = 0.308; FF-el: *F*(2,17) = 0.675 *p* = 0.522].

## 4. Discussion

In the present study, preserved residual hearing after deep intracochlear application of triamcinolone-acetonide via the inner ear catheter and subsequent cochlear implantation was observed in 40% of cases. In addition, 30% of the patients showed only a moderate PTA shift between 15 and 30 dB. The remaining 30% of the patients showed hearing loss of more than 30 dB. The overall median hearing loss was 20.6 dB. The results are similar to the results from our previous study investigating 120 patients with residual hearing treated with flexible lateral wall electrode arrays (Suhling et al., 2016): The median hearing loss was 24.0 dB for the MED-EL FLEX28 electrode arrays (Suhling et al., 2016). The median hearing loss after implantation with shorter electrode arrays was 17.5 dB for the MED-EL FLEX20 and 20.0 dB for the MED-EL FLEX24 electrode arrays (Suhling et al., 2016). A systematic literature review in hearing preservation rates with medium (MED-EL FLEX24) and longer (MED-EL FLEX28) flexible lateral wall electrode arrays showed no difference between medium-length (93.4–93.5%) and longer (92.1–86.8%) electrodes at 4 months (*p* = 0.689) and at 12 months (*p* = 0.219) after implantation (Van de Heyning et al., 2022). In this study, the HEARRING criteria were used to calculate the hearing preservation rates; however, only patients with complete and partial hearing preservation were considered in the final numbers, but not patients with minimal hearing preservation (Van de Heyning et al., 2022). Based on the results from this systematic literature review (Van de Heyning et al., 2022) and from studies investigating lateral wall electrodes and hearing preservation (Wanna et al., 2014; O’Connell et al., 2016; Pillsbury et al., 2018; Shew et al., 2021), lateral wall electrodes are considered to be less traumatic and the optimal electrode array for patients with residual hearing. The fact that the hearing loss after catheter-based deep intracochlear steroid application and after implantation with the FLEX28 electrode array is quite similar or even less when compared to the use of the same lateral wall electrode array without the use of the catheter in terms of hearing preservation is an important clinical observation due to two reasons. Firstly, the use of the longest possible atraumatic electrode arrays shows the best results in hearing performance even if the residual hearing is lost over time (Rajan et al., 2018). Secondly, preserved residual hearing especially in the lower frequencies leads to improved speech perception with the cochlear implant even without the use of a hearing aid in combination with electrical stimulation (Kant et al., 2022). Thus, combining long lateral wall electrode arrays with catheter-based local drug delivery of a variety of pharmaceutical agents and even cell-based therapeutics might present an interesting feature for hearing preservation cochlear implantation approaches.

Hearing preservation rates after intravenous dexamethasone application in an Irish study including more than 70 ears treated with a Cochlear™ or Advanced Bionics™ device with straight and perimodiolar electrode arrays were described as complete when 25% of the residual hearing was lost (in 13% of the patients), as partial up to 75% of the hearing was lost (in 39.1% of the patients) and minimal when more than 75% of the residual hearing was lost (in 30.4% of the patients) according to the HEARRING criteria up to 1 year after implantation. Complete loss of hearing (no detection of residual hearing at all) was observed in 17.4% of the adult population (Gendre et al., 2022). In the pediatric population (mean age at implantation 9.5 years; range 2.25 to 17.33 years) of the same study, better hearing preservation rates were observed (complete 20.7%, partial 51.7% and minimal 13.8% hearing preservation; complete loss of hearing in 13.8%) (Gendre et al., 2022). When looking at the graph showing the averaged PTA shift 3 months after surgery in the low frequencies in the pediatric population, a hearing loss of more than 30 dB was obvious (Gendre et al., 2022). This is in the range of the results from a clinical investigation on 203 patients with residual hearing and the treatment with slim modiolar electrode arrays, which showed a mean hearing loss of 25.9 ± 16.2 dB and a tip fold-over in 7.4% of the patients (Shew et al., 2021).

Cochlear pharmacokinetics may be different in the implanted cochlea as has been shown in a guinea pig model (Salt and Hirose, 2018). The volume and intracochlear flow generated through the cochlear aqueduct, a connection between the perilymphatic and cerebrospinal fluid space, is very low when the cochlea is sealed properly (Salt et al., 2017). By contrast, the perilymph is quickly replaced by CSF when the otic capsule is perforated (Salt and Hirose, 2018). Whether these observations apply in humans is unclear since the human cochlear aqueduct is longer and narrower than in rodents (Burton et al., 2019). Thus, the influence of CSF entry on the pharmacokinetics of the cochlea may be neglectable in humans (Manrique-Huarte et al., 2021).

One major concern with the application of intracochlear steroids via a catheter is an increase in intracochlear pressure leading to microtrauma, inflammation, fibrosis formation and loss of residual hearing. The herein presented results of hearing preservation after the use of the catheter for deep intracochlear steroid injection could be one indicator against such microtraumata. However, a direct measure for cochlear microtrauma does not exist and therefore, the presence or absence of microtraumata cannot be proven. Using the catheter, we applied triamcinolone-acetonide that can - due to its lipophilic attributes - pass anatomic boundaries more readily than dexamethasone (Salt et al., 2019; Salt and Plontke, 2020). However, the only available formulation for injections that we used contains also benzyl alcohol as solvent that could potentially harm delicate cells. For example, investigations on cultured retinal pigment epithelial cells showed that a clinically relevant concentration of 0.225 mg/mL of benzyl alcohol contained in triamcinolone-acetonide formulations that are injected intravitreally caused ultrastructural damage leading to necrosis (Chang et al., 2008). Whether these concentrations are sufficient to cause significant damage to inner ear cells has not been investigated thus far. The use of formulations of triamcinolone-acetonide without solvents and preservatives if available should be therefore recommended for intracochlear application.

There are several limitations associated with this study. Only a limited number of patients with residual hearing were treated with the inner ear catheter and included in this retrospective analysis. The follow up period of the patients reported and analyzed in this study is rather short limiting the strength of the findings. In addition, the amount of the drug and the pace of drug instillation was not controlled in this study and therefore considerable differences may exist that could influence the results. Despite the fact that considerable data on drug distribution, clearance and metabolism exist from preclinical experiments, such information after drug application to the human cochlea is not available hitherto and cannot be taken into account in the discussion of the results. Other factors that could influence hearing preservation such as age, underlying disease, individual cochlear anatomy and surgical expertise have not been controlled in the present study. Despite these limitations, our study is -to our knowledge- the first and only to demonstrate the feasibility of catheter-based application of glucocorticoids in human cochlear implantation.

Despite the presence of benzyl alcohol, the degree of hearing preservation that was achieved after the use of deep intracochlear injections of triamcinolone-acetonide via the inner ear catheter is similar to the one achieved in our previous study (Suhling et al., 2016). This supports our assumption that fluid application to the cochlea via the catheter does not damage the organ of Corti. Thus, our results encourage the use of the inner ear catheter to apply drugs into the cochlea alongside cochlear implantation with the attempt to preserve residual hearing. Due to the development of numerous molecularly defined substances with distinct mechanisms of action, the pharmacotherapy of the inner ear will play an important role in the prevention and treatment of acute and progressive sensorineural hearing loss in the near future (Schilder et al., 2019; Warnecke et al., 2022). In combination with cochlear implantation, the regeneration of neuronal structures, the suppression of the body’s own foreign body reaction and the preservation of residual hearing can substantially improve the success of the therapy. Established local drug delivery for the inner ear is currently realized via the round window membrane. Varying size of the membrane, limited permeability, bony overhang and poor diffusion properties make it difficult to control how much of the substance diffuses into the cochlea (Plontke et al., 2016, 2017; Plontke et al., 2022). The inner ear catheter is safe for intracochlear structures as shown by at least equal hearing preservation rates compared to the treatment with a CI electrode alone in this study. Therefore, it is a suitable application method with which defined amounts of a substance can be delivered to their defined site of action. Furthermore, the inner ear catheter is not only suitable for the application of drugs. Extracellular vesicles have also been successfully applied (Warnecke et al., 2021, 2022) and it might also be an option for gene and cell therapies that are emerging in the future.

Based on the prototype discussed in this publication, the Inner Ear Catheter was CE-marked (INCAT from MED-EL GmbH) and is available for clinical application in the EU and several other countries.

## Data availability statement

The original contributions presented in the study are included in the article/supplementary material, further inquiries can be directed to the corresponding author.

## Ethics statement

Ethical review and approval was not required for the study on human participants in accordance with the local legislation and institutional requirements. The patients/participants provided their written informed consent to participate in this study.

## Author contributions

NP, TL, and AW: conception or design of the work. NP, TL, AW, and RS: acquisition of data. LG and AL-S: analysis and interpretation of data. AW: drafting the work. NP, RS, TL, LG, and AL-S: revising the manuscript critically for important intellectual content. All authors provided approval for publication of the content and agreed to be accountable for all aspects of the work in ensuring that questions related to the accuracy or integrity of any part of the work are appropriately investigated and resolved.

## Funding

This work was supported by the German Research Foundation, Cluster of Excellence “Hearing 4 All” (DFG Exc. 2177), Hanover, Germany. NP and AW received travel grants from MED-EL.

## Conflict of interest

The authors declare that the research was conducted in the absence of any commercial or financial relationships that could be construed as a potential conflict of interest.

## Publisher’s note

All claims expressed in this article are solely those of the authors and do not necessarily represent those of their affiliated organizations, or those of the publisher, the editors and the reviewers. Any product that may be evaluated in this article, or claim that may be made by its manufacturer, is not guaranteed or endorsed by the publisher.
